# Lipoteichoic Acid in *Streptomyces hygroscopicus*: Structural Model and Immunomodulatory Activities

**DOI:** 10.1371/journal.pone.0026316

**Published:** 2011-10-18

**Authors:** Marlène Cot, Aurélie Ray, Martine Gilleron, Alain Vercellone, Gérald Larrouy-Maumus, Elise Armau, Sophie Gauthier, Gérard Tiraby, Germain Puzo, Jérôme Nigou

**Affiliations:** 1 CNRS, IPBS (Institut de Pharmacologie et de Biologie Structurale), Toulouse, France; 2 Université de Toulouse, UPS, IPBS, Toulouse, France; 3 Cayla InvivoGen, Research Department, Toulouse, France; Hopital Raymond Poincare - Universite Versailles St. Quentin, France

## Abstract

Gram positive bacteria produce cell envelope macroamphiphile glycopolymers, i.e. lipoteichoic acids or lipoglycans, whose functions and biosynthesis are not yet fully understood. We report for the first time a detailed structure of lipoteichoic acid isolated from a *Streptomyces* species, i.e. *Streptomyces hygroscopicus subsp. hygroscopicus* NRRL 2387T. Chemical, MS and NMR analyses revealed a polyglycerolphosphate backbone substituted with α-glucosaminyl and α-N-acetyl-glucosaminyl residues but devoid of any amino-acid substituent. This structure is very close, if not identical, to that of the wall teichoic acid of this organism. These data not only contribute to the growing recognition that lipoteichoic acid is a cell envelope component of Gram positive Actinobacteria but also strongly support the recently proposed hypothesis of an overlap between the pathways of lipoteichoic acid and wall teichoic acid synthesis in these bacteria. *S. hygroscopicus* lipoteichoic acid induced signalling by human innate immune receptor TLR2, confirming its role as a microbe-associated molecular pattern. Its activity was partially dependant on TLR1, TLR6 and CD14. Moreover, it stimulated TNF-α and IL-6 production by a human macrophage cell line to an extent similar to that of *Staphylococcus aureus* lipoteichoic acid. These results provide new clues on lipoteichoic acid structure/function relationships, most particularly on the role of the polyglycerolphosphate backbone substituents.

## Introduction

Gram positive cell envelopes are characterized by the presence of cell-wall glycopolymers that are attached either to peptidoglycan or to membrane lipids. Lipid-linked glycopolymers are referred to as macroamphiphiles [1], [2]. Many species contain both types of them [3]–[5]. The main macroamphiphiles are lipoteichoic acids (LTA), which are encountered in the majority of low G+C bacteria (Firmicutes) [6], [7]. LTA is composed of a lipid anchor linked to a chain of poly-glycerol or poly-ribitol units separated by a phosphate group. Such repeated units are also characteristic of teichoic acids linked to peptidoglycan. In contrast, some of the high G+C Gram positive bacteria, i.e. Actinobacteria, do not produce LTA but rather lipoglycans [3], [8]–[11]. Lipoglycans, of which mycobacterial lipoarabinomannan (LAM) is the archetype molecule [12], are macroamphiphiles made of a long chain of mannosyl units with possible arabinose and/or mannose ramifications. They were proposed to discriminate bacteria of the phylum Actinobacteria [3], [7]. However, LTA molecules have recently been characterized in a few number of Actinomycetes, such as *Agromyces* species [13] and *Thermobifida fusca* [1] and several lines of evidence suggest that *Streptomyces* species would also produce LTA [14], [15]. Identification of LTA in Actinobacteria raises the question of its biosynthesis in these bacteria. Indeed, the enzyme LtaS polymerase, which catalyses phospho-glycerol unit polymerization and is essential for LTA synthesis in *Staphylococcus aureus*, lacks clear orthologues in the sequenced actinobacterial genomes [2]. Thus, LTA biosynthesis in Actinomycetes may proceed by an alternative pathway and Sutcliffe and colleagues [2] propose the attractive hypothesis that it could be achieved by a pathway that overlaps with that of teichoic acid biosynthesis, as suggested recently for *Streptococcus pneumoniae* [16].

The physiological roles of LTA are not yet fully understood. LTA is indispensable in *S. aureus*, however its amount can be reduced by 90% with only minor phenotypic changes during *in vitro* cultivation [17]. It seems to play a role in cell division, bacterial surface properties, autolysin activity and cation storage [5], [17], [18]. Interestingly, LTA expression on the group B *Streptococcus* surface plays a role in bacterial interaction with blood-brain barrier endothelium and the pathogenesis of neonatal meningitis [19]. However, the role of LTA in infection and inflammation is somewhat controversial. For some authors, LTA shares with LPS many of its pathogenic properties [20], [21] and is thought to be involved in infection and post infection sequelae, such as septic shock caused by Gram-positive bacteria [20]. On the contrary, others argue that LTA is not the dominant immunobiologically active compound in *S. aureus* [22]. LTA, as well as lipoglycans, are recognized by the innate immune system *via* Toll-like receptor 2 (TLR2), a receptor that plays a crucial role in detecting invading Gram-positive bacteria. TLR2-stimulating activity of purified LTA has been a matter of controversy, contamination of LTA fractions by highly active lipopeptides being formally difficult to rule out [23]. However, immune activation is induced by synthetic LTA [24] and by LTA from a mutant *S. aureus* strain lacking lipoproteins [25], confirming the role of LTA as a microbe-associated molecular patterns (MAMPs) of Gram-positive bacteria detected by the innate immune system.

In the present study, we report for the first time a detailed structural model of LTA isolated from a *Streptomyces* species. Its capacity to induce TLR2 signaling and to stimulate cytokine production was investigated. Altogether, our results provide new clues on LTA biosynthesis in Actinobacteria and LTA structure/function relationships.

## Results

### Extraction, purification and structural characterization of a LTA from S. hygroscopicus NRRL 2387 (ShLTA)

**Figure 1:**
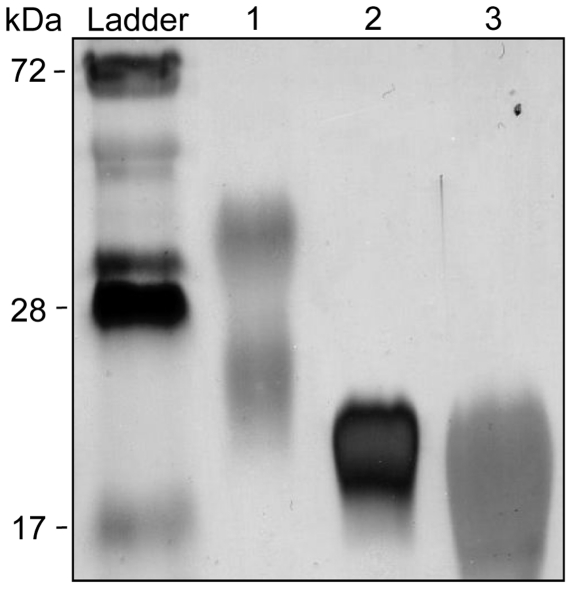
SDS-PAGE analysis of a macroamphiphile glycopolymer from *S. hygroscopicus*. Lane 1, *Mycobactrium tuberculosis* LAM and LM (top and bottom bands, respectively); lane 2, *S. hygroscopicus* macroamphiphile glycopolymer; lane 3, *S. aureus* LTA. The gel was revealed by periodic acid-silver nitrate staining. LAM, lipoarabinomannan; LM, lipomannan; LTA, lipoteichoic acid.

**Figure 2:**
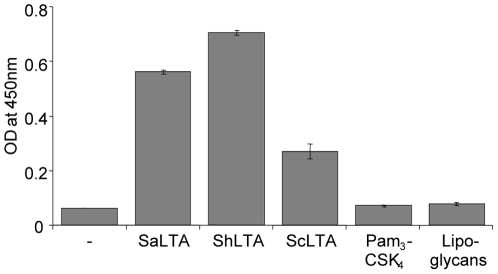
An anti-LTA antibody recognizes *S. hygroscopicus* macroamphiphile glycopolymer. 100 ng of *S. hygroscopicus* or *S. coelicolor* macroamphiphile glycopolymers (ShLTA and ScLTA, respectively), *S. aureus* LTA (SaLTA), Pam_3_CSK_4_ synthetic lipopeptide or *M. tuberculosis* lipoglycans mixture were coated in microtiter plate wells and probed with an antibody directed against *S. epidermis* LTA (anti-LTA). The results are mean ± SD and are representative of three separate experiments.

Macroamphiphile glycopolymers are classically extracted by a hot phenol-water procedure [26]. However, this might result in partially degraded LTA and most particularly in the loss of alanine substituents [27]. *S. hygroscopicus* NRRL 2387 cells were thus extracted by the more gentle butanol procedure introduced by Morath *et al.* [27]. After enzymatic degradation of the nucleic acid and protein contaminants, the fraction was purified by hydrophobic interaction chromatography (HIC). This allowed removal of a glycopolymer, eluted with 10% isopropanol, composed of galactose and KDN (2-keto-3-deoxy-D-glycero-D-galacto-nononic acid) previously described in *Streptomyces* species [28], [29] and that represented around 50% (w/w) of the fraction before HIC. SDS-PAGE analysis of the HIC fraction eluted with 35% of isopropanol revealed a compound with an apparent molecular weight of around 20 kDa and a migration pattern, as a broad band, similar to that of mycobacterial lipoglycans or LTA from *S. aureus* (SaLTA) (Figure 1). Its molecular mass distribution was estimated by MALDI-TOF mass spectrometry to be between 7 and 9 kDa for the major molecular species (not shown). Chemical analyses of the compound indicated a mean phosphorus content of 20 mol of phosphorus per mol of molecule, suggesting a LTA rather than a lipoglycan structure. Accordingly, it was recognized in ELISA experiments by an antibody directed against *Staphylococcus epidermis* LTA (Figure 2). This antibody also recognized SaLTA but neither mycobacterial lipoglycans nor the synthetic lipopeptide Pam_3_CSK_4_. In addition, glycerol (Gro) and glycerol-phosphate (Gro-P) were detected by gas chromatography (GC)/MS suggesting a polyglycerolphosphate LTA (PGP-LTA). The compound was subsequently termed ShLTA. The predominant fatty acids detected by GC after alkaline hydrolysis were C16:0 (40%), isoC16 (11%), isoC17:0 (13%), anteisoC17:0 (28%), isoC15:0 (2%) and anteisoC15:0 (4%), a profile typical of *Streptomyces* species whole cells [30]. Glucosamine (GlcN) but neither mannosamine nor galactosamine were detected by GC and capillary electrophoresis monitored by laser-induced fluorescence (CE-LIF) after strong acid hydrolysis of ShLTA. Quantitative analyses by colorimetric assays indicated a ratio GlcN/phosphorus of 0.28/1 (mol∶mol) (Table 1). No release of amino acids (<0.1% w/w), after acid hydrolysis, could be detected by a highly sensitive approach based on liquid chromatography (LC) monitored by LIF [31], indicating that ShLTA is neither substituted by amino acids nor contaminated by traces amount of lipopeptides.

**Table 1:** Relative abundance of phosphorus, glycerol and glucosamines in *S. hygroscopicus* LTA as determined by biochemical and NMR analyses.

	Biochemical^a^	NMR^b^
P	1	-
Gro:	1.7	1:
(P-1-Gro-3-P)		(0.50)
(P-1-Gro-3-P)		(0.22)^c^
|		
GlcN		
(P-1-Gro-3-P)		(0.06)^c^
|		
GlcNAc		
(P-2-Gro-3-P)		(0.22)
GlcN+GlcNAc	0.28	0.28

a Relative quantification of P and amino sugars (i.e. GlcN+GlcNAc) was performed by colorimetric assays, the amount of P being fixed to 1. The ratio Gro/amino sugars was determined by GC but is overestimated because of partial amino sugar degradation during strong acid hydrolysis.

b Relative quantification of the different Gro derivatives was performed by integration of the corresponding H-2/C-2 cross peak on ^1^H-^13^C HMQC spectrum (Figure 3G).

c These values perfectly fit with the GlcN/GlcNAc ratio of 3.7/1 as determined by integration of the corresponding anomeric signals (Figure 3D).

Gro, glycerol; GlcNAc, N-acetyl-glucosamine; GlcN, glucosamine; P, phosphate; P-1-Gro-3-P, P-2-Gro-3-P, 1,3- and 2,3-diphospho-glycerol units respectively.

To get further insights into the distribution of the different structural motifs along the molecule backbone, ShLTA was submitted to 48% hydrofluoric acid (HF) hydrolysis for 48 h at 4°C, a reaction known to hydrolyze phosphodiester bonds. After a chloroform/methanol/water partition, the aqueous phase was analyzed by MALDI-TOF/MS and MS/MS. The data are summarized in Table 2. HF treatment did not completely hydrolyse all the phosphodiester bonds. Indeed, the positive MALDI mass spectrum showed peaks at *m/z* 254.1, 336.1, 408.1, 569.2 and 611.2 attributed to (M+H)^+^ molecular ions that could be assigned to structures containing the motifs Gro-GlcN, P-Gro-GlcN, Gro-P-Gro-GlcN, Gro-GlcN-P-Gro-GlcN, and Gro-GlcNAc-P-Gro-GlcN respectively (Table 2). The assignments were confirmed by MS/MS analysis showing fragment ions characteristic of the loss of an amino sugar (i.e. GlcN) at *m/z* 162.0 or a N-acetylated amino sugar (i.e. GlcNAc: N-acetyl-glucosamine) at *m/z* 204.0 [32] (Table 2). Interestingly, the molecular ion at *m/z* 611.2 corresponded to a fragment containing both N-acetylated and non-N-acetylated amino sugars, suggesting that the PGP backbone of ShLTA was substituted by both types of amino sugars. The control that a GlcNAc standard was not hydrolyzed into GlcN by HF treatment supported the above hypothesis.

**Table 2:** Positive ion MALDI-TOF/MS and MS/MS analyses of structural motifs generated by HF hydrolysis (48%, 48 h at 4°C) of ShLTA.

*MS* Parent ions *m/z*			
(relative intensity)	*MS/MS* Fragments ions *m/z*	Composition M	Molecular formula M
162.0 (M^+^) (1)	-	Anhydro-GlcN	C_6_H_12_O_4_N
204.0 (M^+^) (0.21)	-	Anhydro-GlcNAc	C_7_H_14_O_5_N
254.1 (M+H^+^) (0.97)	162	Gro-GlcN	C_9_H_19_O_7_N
336.1 (M+H^+^) (0.11)	162	P-Gro-GlcN	C_9_H_22_O_10_NP
408.1 (M+H^+^) (0.28)	162, 247^a^	Gro-P-Gro-GlcN	C_12_H_26_O_12_NP
569.2 (M+H^+^) (0.15)	162, 247^a^	Gro-GlcN-P-Gro-GlcN	C_18_H_37_O_16_N_2_P
611.2 (M+H^+^) (0.14)	162, 204, 408, 450^a^	Gro-GlcNAc-P-Gro-GlcN	C_20_H_39_O_17_N_2_P

*m/z* of parent and fragment ions, possible composition and Molecular formula are shown. Gro, glycerol; GlcNAc, N-acetyl-glucosamine; GlcN, glucosamine; P, phosphate group.

a loss of GlcN.

Native ShLTA was subsequently analyzed by NMR. The ^1^H-NMR anomeric region exhibited two anomeric signals at δ 5.43 (GI_1_) and δ 5.10 (GII_1_) in a ratio 3.7/1 (Figure 3D), tentatively attributed to both types of glucosamines (i.e. GlcN and GlcNAc). As revealed by ^1^H-^13^C HMQC spectrum, their corresponding anomeric carbons resonate at δ 96.0 (GI_1_) and δ 98.3 (GII_1_) (Figure 3F). Proton and carbon resonances of both spin systems were assigned from ^1^H-^13^C HMQC and ^1^H-^1^H HOHAHA experiments (partially shown in Figures 3F, 3G and 3E, respectively). The assignments are summarized in Table 3. The chemical shift of C2 in both spin systems around 55 ppm was typical of amino sugars and the ^1^ *J*_C1,H1_ coupling constant of 174 Hz indicated an α-anomeric configuration for both units. H2 resonances at δ 3.36 (GI_2_) and δ 3.94 (GII_2_) (Figure 3E) were indicative of a non-N-acetylated and an N-acetylated glucosamine, respectively [33]. This was confirmed by a NOESY experiment recorded with ShLTA dissolved in H_2_O/D_2_O (9∶1, v/v) (Figures 3B and C). Indeed, an amide proton at δ 8.21 (GII_NH_) (Figure 3A) showed intracyclic correlations with different protons that belonged to spin system GII (Figure 3C). In addition, another correlation was observed with methyl protons of an N-acetyl group at 2.09 ppm (Figure 3B). Altogether the data indicated that GI and GII spin systems correspond to α-GlcN and α-GlcNAc, respectively. Another amide proton resonance at δ 8.29 (GIII_NH_) with very low intensity (<10%) was also observed and most probably corresponded to a minor form of N-acetylated amino sugar, whose characterization could not be carried out further.

**Figure 3:**
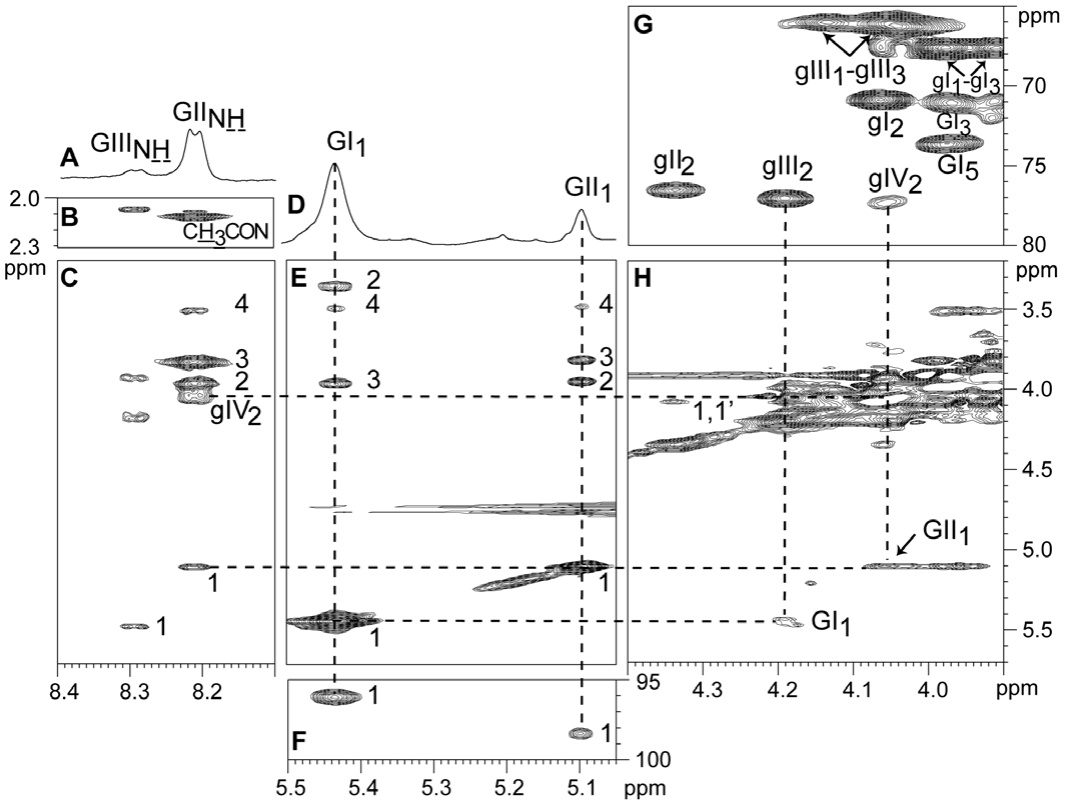
1D ^1^H (A, D), 2D ^1^H-^13^C HMQC (F, G), ^1^H-^1^H HOHOHA τ_m_ 110 ms (E) and ^1^H-^1^H NOESY τ_m_ 500 ms (B, C, H) spectra of ShLTA. ^1^H (D), ^1^H-^13^C HMQC, ^1^H-^1^H HOHOHA and ^1^H-^1^H NOESY (H) were recorded in D_2_O, ^1^H (A) and ^1^H-^1^H NOESY (B, C) in H_2_O/D_2_O (9∶1) at 298K. Expanded regions (δ^1^H: 8.10–8.40) (A), (δ^1^H: 8.10–8.40, δ^1^H: 2.00–2.30) (B), (δ^1^H: 8.10–8.40, δ^1^H: 3.20–5.70) (C), (δ^1^H: 5.05–5.50) (D), (δ^1^H: 5.05–5.50, δ^1^H: 3.20–5.70) (E), (δ^1^H: 5.05–5.50, δ^13^C: 95–100) (F), (δ^1^H: 3.90–4.40, δ^13^C: 65–80) (G) and (δ^1^H: 3.90–4.40, δ^1^H: 3.20–5.70) (H) are shown. GI, α-GlcN, GII, α-GlcNAc, gI to gIV, Gro units (see Table 3 and Figure 4). GIII corresponds to a weakly abundant GlcNAc that could not be further characterized.

**Table 3:** Proton and carbon chemical shifts of *S. hygroscopicus* LTA.

Residue	Name	Protons	δ (ppm)	Carbons	δ (ppm)
P-1)-Gro-(3-P-	gI	H-1, H-1′	3.98/3.91	C-1	67.5
		H-2	4.06	C-2	70.8
		H-3, H-3′	3.98/3.91	C-3	67.5
P-2)-Gro-(3-P-	gII	H-1, H-1′	4.05	C-1	66.8
		H-2	4.33	C-2	76.6
		H-3, H-3′	3.78/3.81	C-3	62.2
P-1)-Gro-(3-P-2)	gIII	H-1, H-1′	4.04/4.13	C-1	66.0
		H-2	4.19	C-2	77.0
|		H-3, H-3′	4.04/4.13	C-3	66.0
α-GlcN	GI	H-1	5.43	C-1	96.0^a^
		H-2	3.36	C-2	55.3
		H-3	3.97	C-3	71.1
		H-4	3.50	C-4	70.9
		H-5	3.96	C-5	73.7
		H-6, H-6′	3.91/3.81	C-6	61.7
P-1)-Gro-(3-P-2)	gIV	H-1, H-1′	3.98	C-1	67.5
		H-2	4.05	C-2	77.3
|		H-3, H-3′	3.90	C-3	67.5
α-GlcNAc	GII	H-1	5.10	C-1	98.3^a^
		H-2	3.94	C-2	55.0^b^
		H-3	3.82	C-3	72.4
		H-4	3.50	C-4	70.9
		H-5	3.97	C-5	73.7
		H-6, H-6′	3.91/3.81	C-6	61.7

Chemical shifts were measured at 298K in D_2_O and are referenced relative to internal acetone signals at δ_H_ 2.225 and δ_C_ 34.00. Gro, glycerol; GlcNAc, N-acetyl-glucosamine; GlcN, glucosamine; P, phosphate; P-1-Gro-3-P, P-2-Gro-3-P, 1,3- and 2,3-diphospho-glycerol units respectively.

a ^1^ *J*_H-1,C-1_ = 174 Hz.

b CH_3_CONH at 2.09 ppm and CH_3_CONH at 8.21 ppm.

Interestingly, nOe contacts were observed between anomeric protons of GI and GII and methine protons at δ 4.19 (gIII_2_) and δ 4.05 (gIV_2_) respectively (Figure 3H), attributed to H2 protons of 1,3-diphospho-glycerol (P-1-Gro-3-P) units (Table 3) [33]. These data indicated that GlcN and GlcNAc units were α-glycosidically linked to the C2 hydroxyl of the PGP repeating units. This was confirmed by an additional correlation on the NOESY spectrum between amide proton GII_NH_ at δ 8.21 and proton gIV_2_ (Figure 3D) and by HMBC experiment showing connectivities between anomeric protons of GI and GII and C2 of gIII and gIV units at δ 77.0 (gIII_2_) and δ 77.3 (gIV_2_) respectively (not shown) (Table 3). The deshielding of the C2 resonances of these units (gIII_2_ and gIV_2_) as compared with C2 resonance of an unsubstituted P-1-Gro-3-P unit at δ 70.8 (gI_2_) (Δδ 6.2 and 6.5 ppm, respectively) (Figure 3G; Table 3) was also in agreement with their substitution by glycosyl units. In contrast, C2 of Gro unit gII (gII_2_), whose resonance at δ 76.6 was also deshielded as compared to that of gI (Δδ 5.8 ppm) (Figure 3G; Table 3), did not show in the HMBC experiment any correlation with protons, except with those of its own spin system (not shown). Similarly, H2 of gII unit did not correlate in NOESY or ROESY experiments with any protons other than those of its own spin system. Although clearly not glycosylated, the deshielding of C2 resonance indicated that this position was substituted and we hypothesized that gII spin system corresponded to 2,3-diphospho-glycerol (2-P-Gro-3-P) units, as previously described [34]. This was confirmed by ^31^P NMR analysis of deacylated ShLTA. Indeed, on ^1^H-^31^P HMQC spectrum, a phosphate resonance at 2.3 ppm correlated with H2 of gII unit at 4.33 ppm (not shown), indicating that the C2 hydroxyl of gII was directly substituted by a phosphate group.

**Figure 4:**
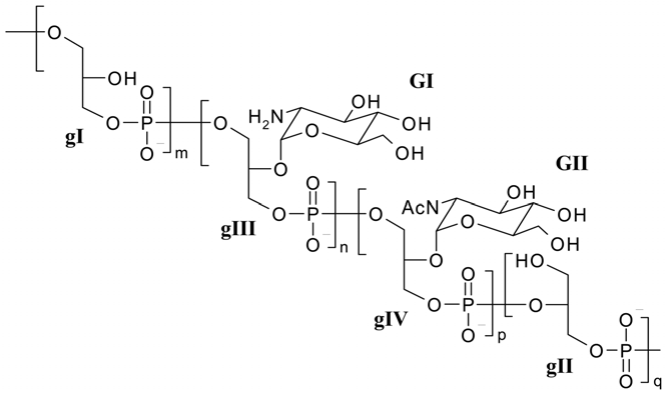
Proposed structure of ShLTA. The PGP backbone is shown, with n, m, p and q estimated to be ca. 10, 4, 1 and 4 respectively. The relative position of the different motifs along the backbone is unknown. The LTA lipid anchor, presumptively a glycosyl-containing diacylglyceride glycolipid, is not shown. GI, GII and gI to gIV refer to spin systems as defined in Figure 3 and Table 3.

Integration of the Gro unit H2-C2 cross-peaks on HMQC experiment (Figure 3G) allowed us to obtain the relative proportion of the different motifs on the PGP chain (Table 1) and propose the structural model depicted in Figure 4.

### TLR2 activation and cytokine production

**Figure 5:**
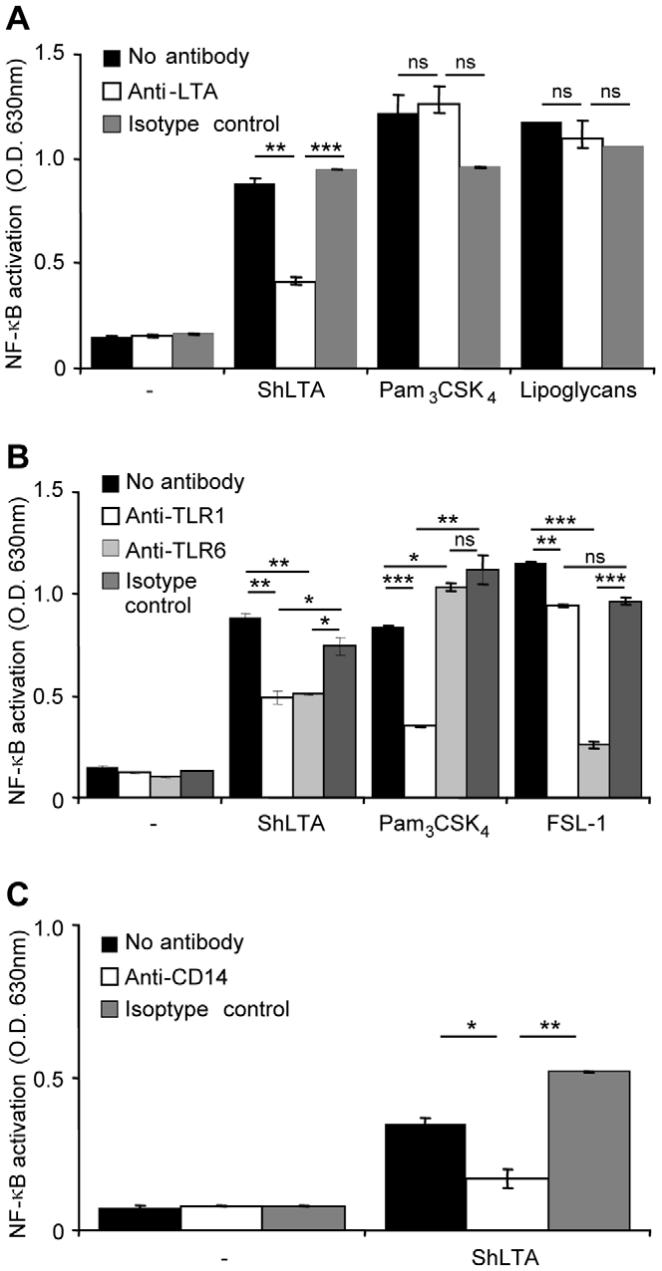
ShLTA activity is inhibited by an anti-LTA antibody and is dependent on TLR1, TLR6 and CD14. A. Stimuli were pre-incubated for 30 min at 37°C with 5 µg.ml^−1^ of anti-LTA or an IgG1 isotype control before HEK-TLR2 cells addition. ShLTA, Pam_3_CSK_4_ and *M. tuberculosis* lipoglycans were tested at a concentration of 10 ng.ml^−1^. B, C. HEK-TLR2 cells were pre-incubated for 30 min at 37°C, before stimuli addition, with 5 µg.ml^−1^ of various monoclonal antibodies: anti-TLR1 and anti-TLR6 (B), anti-CD14 (C) or IgG1 isotype control. ShLTA was tested at a concentration of 10 ng.ml^−1^. Pam_3_CSK_4_ (5 ng.ml^−1^) and FSL-1 (0.5 ng.ml^−1^) were used as positive controls of TLR2/TLR1 and TLR2/TLR6 agonists, respectively. NF-κB activation was determined by reading OD at 630 nm. The results are mean ± SD and are representative of three separate experiments. *, P<0.05; **, P<0.01; ***, P<0.001; ns, not significant.

**Figure 6:**
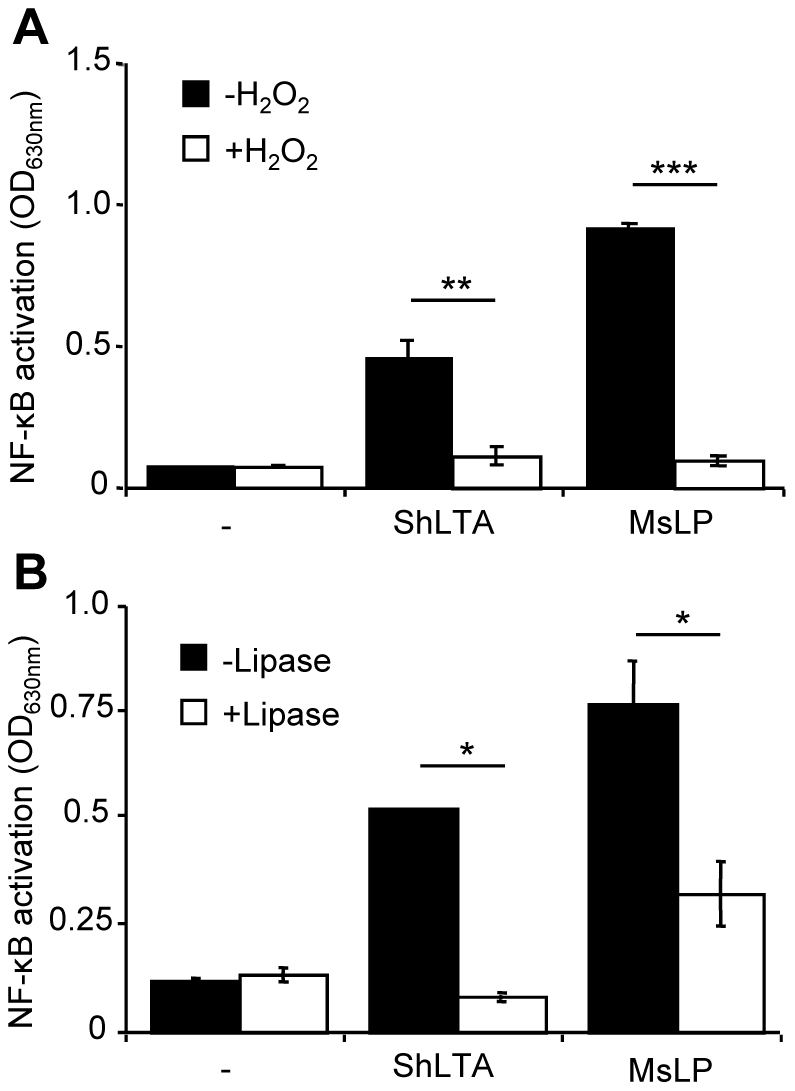
H_2_O_2_ (A) or lipoprotein lipase (B) treatments alter ShLTA capacity to stimulate TLR2. HEK-TLR2 cells were stimulated with 10 ng.ml^−1^ ShLTA or *M. smegmatis* lipoproteins (MsLP) previously treated or not with 1% H_2_O_2_ for 24 h at 37°C (A) or with *Pseudomonas sp.* lipoprotein lipase (B). NF-κB activation was determined by reading OD at 630 nm. The results are mean ± SD and are representative of three separate experiments. *, P<0.05; **, P<0.01; ***, P<0.001.

**Figure 7:**
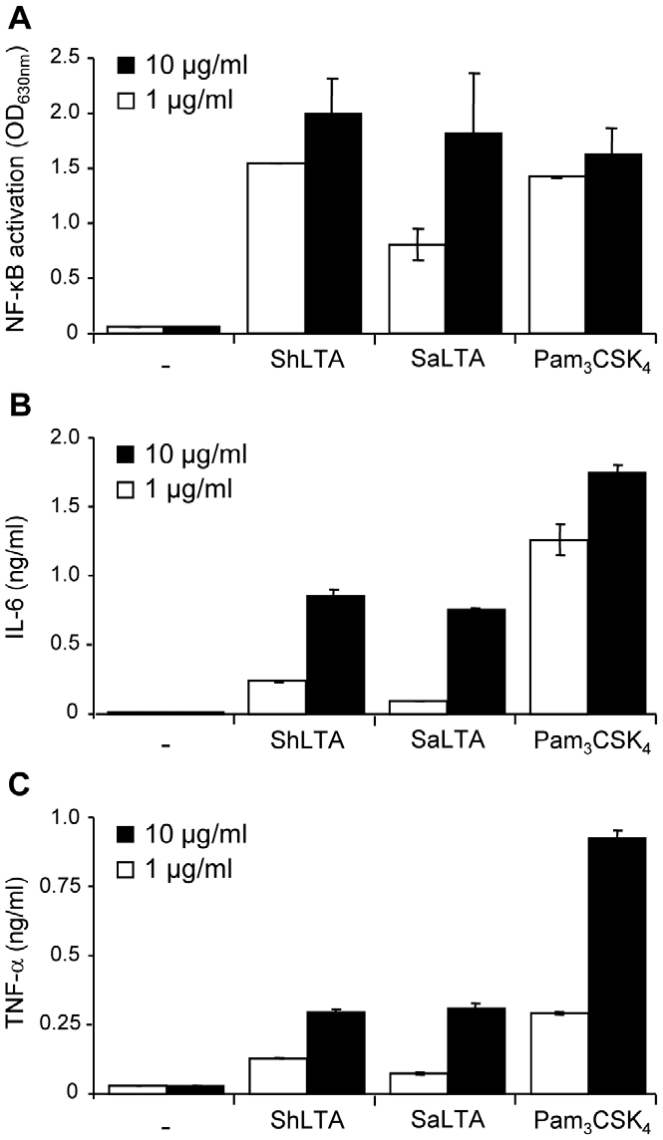
ShLTA stimulates NF-κB activation (A) and IL-6 (B) and TNF-α (C) production by human THP-1 monocyte/macrophage cell line. A. THP-1 cells were stimulated with 1 or 10 µg.ml^−1^ of ShLTA, SaLTA or Pam_3_CSK_4_. NF-κB activation was determined by reading OD at 630 nm. B, C. THP-1 cells were differentiated with 20 ng.ml^−1^ of PMA for 24 h and then stimulated with 1 or 10 µg.ml^−1^ of ShLTA, SaLTA or Pam_3_CSK_4_. IL-6 and TNF-α were assayed in the supernatant by sandwich ELISA. The results are mean ± SD and are representative of three separate experiments.

LTA has been reported to activate phagocytic cells *via* recognition by TLR2 and TLR6 [35]–[38]. The molecular bases of this recognition have been recently partially uncovered by the resolution of a crystal structure of a soluble form of TLR2 in complex with *S. pneumonia* LTA [39]. We first tested the ability of ShLTA to stimulate HEK293 cells stably transfected with human TLR2 and CD14 genes and a NF-κB-inducible reporter system (HEK-TLR2 cells). As expected, ShLTA induced NF-κB activation in HEK-TLR2 cells (Figure 5) but not in the parent HEK cells (not shown). ShLTA stimulatory activity was inhibited by the anti-LTA antibody (Figure 5A). The latter did not show any effect on activation by the TLR2 ligands Pam_3_CSK_4_ (Figure 5A) or FSL-1 (not shown), strongly suggesting that ShLTA activity was not due to contaminating lipopeptides. However, in agreement with a recent report by Seo *et al.* [40], ShLTA activity was altered by treatment by H_2_O_2_ (Figure 6A) or by a lipoprotein lipase (Figure 6B). Indeed, although previously thought to be selective of lipopeptide/lipoproteins [22], [23], these treatments have been recently shown to also affect the chemical structure of pneumococcal and staphylococcal LTA [40]. The requirement of TLR1 or TLR6 for recognition of ShLTA by TLR2 was tested using blocking antibodies. Whereas the activity of the triacylated Pam_3_CSK_4_ and diacylated FSL-1 lipopeptides was clearly dependant on TLR1 and TLR6 respectively, activity of ShLTA was partially inhibited by both anti-TLR1 and anti-TLR6 antibodies (Figure 5B). ShLTA activity was also dependant on CD14 (Figure 5C). Finally, we investigated the capacity of ShLTA to activate the human THP-1 monocyte/macrophage cells, using a cell line derivative that stably expresses a NF-κB-inducible reporter system. ShLTA induced NF-κB activation (Figure 7A) and IL-6 (Figure 7B) and TNF-α (Figure 7C) production by these cells to an extent similar to that of SaLTA.

## Discussion

In the present study, we report the first detailed structure of a LTA isolated from a *Streptomyces* species, i.e. *S. hygroscopicus*, and so confirms the presence of LTA in *Streptomyces* as previously suggested [14], [15]. The assumption that LTA was absent from Actinomycetes most probably resulted from a sampling bias, since essentially families from the suborder *Corynebacterineae*, that all contain lipoglycans, have been analyzed so far [1]. In agreement with a preliminary report by Rahman *et al.* [15], we also isolated LTA in *Streptomyces coelicolor* (ScLTA). It was recognized by the anti-LTA antibody (Figure 2) and preliminary structural analyses indicated that this LTA, as ShLTA, was of PGP type. However, surprisingly, we could not detect any macroamphiphile glycopolymer in *Streptomyces verticilus*. Similarly, Rahman *et al.* reported the apparent absence of macroamphiphiles in the thermophilic Actinomycete *Rubrobacter xylanophylus* [1]. Our data thus extend their observation and demonstrate that within a given genus, some species can contain LTA and others not. This phenomenon has not been reported so far in lipoglycan-containing genera.

ShLTA was found to contain a PGP backbone, composed of 78% of 1-P-Gro-3-P and 22% of 2-P-Gro-3-P repeating units. A portion of the 1-P-Gro-3-P units were α-glycosylated at position 2 by GlcN or GlcNAc (Figure 4). Not unexpectedly, ShLTA was devoid of D-Alanine substituents found in many PGP-LTA. Indeed, this result is consistent with the fact that the genomes of Actinobacteria, including *Streptomyces spp.*, lack orthologues of the dlt system, which is dedicated to incorporation of D-Alanine into PGP-LTA and teichoic acids [1]. Interestingly, the PGP structure of shLTA is very similar, apart for lacking *O*-acetylation, to that of the *S. hygroscopicus* strain ISP 5578 ( = NRRL 2387T) wall teichoic acid previously described by Tul'skaia *et al.* [34]. This contrasts with many Firmicutes, such as *S. aureus* or *B. subtilis*, for which LTA and teichoic acids have unrelated structures and are synthesized by two distinct pathways [3], [41]. The genomes of Actinobacteria sequenced so far (including *S. coelicolor* and other *Streptomyces spp*) and of a few Firmicutes, such as *S. pneumoniae*, lack clear orthologues of the gene encoding LtaS polymerase [1]. This enzyme catalyses phospho-glycerol unit polymerization of LTA and is essential for its synthesis in *S. aureus*. Consequently, it has been suggested that in *S. pneumoniae*, LTA synthesis could be achieved by an alternative pathway that overlaps with that of teichoic acid biosynthesis [16], [42] and Sutcliffe and colleagues [1] propose the attractive hypothesis that this pathway could also take place in Actinomycetes. Our finding that PGP moieties of *S. hygroscopicus* LTA and teichoic acid have a very similar structure further reinforces this hypothesis in which a PGP structure common to both molecules could be synthesized by the same enzymatic machinery and then transferred to peptidoglycan as teichoic acid or to a lipid anchor to yield the PGP-LTA. Accordingly, orthologues of the *B. subtilis* tagB/tagF teichoic acid biosynthesis genes are present in the genome of *S. himastatinicus* (a genome sequenced member of the *S. hygroscopicus* phylogenetic subclade) and other *Streptomyces spp.* (data not shown), supporting the occurrence of a pathway for teichoic acid biosynthesis in these species.

LTA is a powerful Gram-positive immunostimulatory component that induces cytokine production *via* binding to TLR2 on phagocytic cells [43]. The structure/function relationships of this activity are not yet fully understood and possible contamination of LTA fractions by highly stimulatory lipoproteins have been advanced in the literature [44]. However, we found that TLR2 activation by ShLTA could be inhibited by an anti-LTA antibody that had no effect on synthetic lipopeptide activity. Moreover, we failed to detect any amino acids (<0.1% w/w) in the ShLTA fraction used for bioassays, even by the use of a very sensitive technique based on LC-LIF [31]. Altogether, these data suggest that ShLTA is a *bona fide* TLR2 ligand and they are in agreement with the demonstration by Morath *et al.* [24] that synthetic LTA is a potent stimulus of cytokine release. However, in contrast to the proposal by the same authors [24], [27], our data demonstrate that D-alanination is not critical for LTA activity, or at least can be replaced. Indeed, ShLTA, although devoid of D-alanine substituents showed an activity similar to that of SaLTA. The role played by D-alanine in SaLTA activity is not clear. One hypothesis proposed [35] is that they could enable ionic interactions between LTA molecules forming multimers. If this is right, we can imagine that GlcN, rather uncommon in prokaryotes in its non-*N*-acetylated form, as a positively charged substituent, could functionally replace D-alanine in ShLTA. However, it is worth noting that only 22% of the PGP repeating units are substituted by GlcN in ShLTA as compared to 70% by D-alanine in SaLTA [27]. GlcNAc is supposed to have no impact on LTA activity [35]. ShLTA activity was altered by digestion with a lipoprotein lipase, which has been shown to deacylate not only lipoproteins but also LTA [40]. This observation is in agreement with the critical role played by fatty acids for LTA binding to TLR2 [39]. In agreement with a recent report by Seo *et al.* [40], ShLTA activity was also altered by treatment with H_2_O_2_. In contrast to what was previously thought, H_2_O_2_ and lipoprotein lipase treatments are not selective at all of lipopeptide/lipoproteins [22], [23] and cannot be invoked to implicate the latter in TLR2 stimulating activities.

Using blocking antibodies experiments, we found ShLTA activity to be dependent on CD14 and partially on TLR6 and TLR1. Interestingly, these data are in agreement with the recent study by Bunk *et al.* [25] demonstrating the required expression of TLR6 and CD14 for HEK-TLR2 cells activation by SaLTA devoid of lipopeptide contaminants. The choice of the TLR2 heterodimer, with either TLR1 or TLR6, can be dictated by subtle changes in the structure of the TLR2 ligand polar head [39] and is not yet completely understood.

Altogether, our findings contribute to the growing recognition that LTA is a cell envelope component of Gram-positive Actinobacteria [1]. Moreover, the detailed structure of ShLTA PGP moiety strongly supports the hypothesis of an overlap between the pathways of LTA and teichoic acid synthesis in *Streptomyces*. However, this has now to be definitely proved by genetic manipulation. Finally, our results provide new clues on LTA structure/function relationships, most particularly on the role of PGP substituents.

## Materials and Methods

### Srains and growth conditions

*S. hygroscopicus subsp. hygroscopicus* NRRL 2387 (ATCC27438), *S. coelicolor* M145 and *S. verticillus* ATCC 21 890 II6-3 were grown at 27°C for 40 to 50 h under shaking in a culture medium which contained 5 g of yeast extract (BioSpringer), 5 g of bacto soytone peptone (Difco) and 20 g of glucose syrup (Roquette) per liter of deionized water.

### Extraction and purification of LTA

Cells were delipidated at 50°C by mixing with chloroform/methanol (1∶2 and then 1∶1) overnight. After lyophilisation, delipidated cells were resuspended in a 0.1 M citrate buffer pH 4.7 and disrupted by sonication. The lysate was then extracted twice with 1 volume of butan-1-ol. Aqueous phase, containing LTA, was treated with DNAse, RNAse, α-amylase, pronase, α-chymotrypsin and trypsin. After ultrafiltration, samples were purified by hydrophobic interaction chromatography on an octylsepharose CL-4B column eluted with increasing concentrations of isopropanol (10, 15, 20, 25, 35, 40 and 80%) in 0.1 M ammonium acetate. Fractions were collected and analyzed by SDS-PAGE followed by periodic acid-silver nitrate staining. After dialysis and drying, they were submitted to a water/butan-1-ol (1∶1 v/v) partition. The water phases were lyophilized and stored at −20°C. The fraction eluted with 35% isopropanol was used for further analysis.

### H_2_O_2_ and lipoprotein lipase treatments

20 µg of LTA or lipoproteins (lipoproteins were extracted from a cleared lysate of *Mycobacterium smegmatis* by a phenol/water partition as previously described [45]) were resuspended in 100 µl of 1% H_2_O_2_ and incubated at 37°C for 24 h. H_2_O_2_ was evaporated by lyophilisation.

Lipoprotein lipase from *Pseudomonas sp.* (Fluka) was used at a ratio enzyme/LTA or lipoproteins 1∶2 (w/w) in a 100 mM sodium phosphate buffer, 150 mM NaCl, 0.1% Triton X-100, pH 7.2 and incubated for 16 h at 37°C.

### Elisa assay

ShLTA, ScLTA, commercial SaLTA (InvivoGen), *M. tuberculosis* lipoglycan mixture [12], [46] or Pam_3_CSK_4_ (InvivoGen) (100 ng/50 µl of ethanol/water 1∶1, v/v) were adsorbed in 96-wells microtiter plate by air drying and extensively rinsed with washing buffer (TBS, 0.5% BSA, 0.01% Tween 20). Wells were blocked for 2 h at room temperature with TBS, 5% BSA. After washing, 100 µl of 5 µg.ml^−1^ anti-LTA (Abcam, Ab12248) or IgG1 isotype control (eBioscience) antibodies in TBS, 1% BSA were added for 2 h at room temperature. After washing, 100 µl of an HRP-conjugated goat anti-mouse antibody 1/3000 (Sigma) was added in TBS, 1% BSA for 1.5 h at room temperature. HRP activity was detected by addition of 100 µl of Sure Blue TMB substrate (BD Biosciences).

### Chemical analyses

Phosphorus content was determined after treatment of 50 µg LTA by 260 µl of perchloric acid at 170°C for 20 min. Then, 400 µl of freshly prepared solutions of 1.25% molybdate and 5% ascorbate were added. After 5 min boiling in a water bath, samples were cooled in ice. Absorbance was read at 660 nm and phosphorus concentration was calculated relatively to a Na_2_HPO_4_ standard solution.

Amino sugars were analyzed after hydrolysis of 10 µg LTA by 6N HCl at 110°C, overnight. They were detected either by CE-LIF using a 20 mM sodium borate buffer [47] after re-N-acetylation [48] and APTS derivatization [47] or by GC after trimethylsilylation by N,O-bis(trimethylsilyl)trifluoroacetamide/trimethylchlorosilane (99∶1, v/v) in pyridine (1∶1, v/v) for 1 h at 110°C.

Amino sugars were quantified spectrophotometrically after hydrolysis of 200 µg LTA as described above. HCl was evaporated by a nitrogen stream and sample was dissolved in 250 µl of water. Then, 500 µl of a solution of 0.625 M sodium carbonate/acetylacetone (48∶2, v/v) were added. After 10 min boiling in a water bath, the solution was cooled and 2.5 ml of 95% ethanol were added. After 5 min at 75°C, 500 µl of 5.3% *p*-dimethylaminobenzaldehyde in 37% HCl were added and the solution was incubated again for 30 min at 75°C. 2.5 ml of 95% ethanol were further added and after 30 min, OD was read at 520 nm. Amino sugar concentration was calculated relatively to a GlcN standard solution.

Amino acids were analyzed after hydrolysis of 100 µg LTA by 6N HCl at 110°C, overnight by LC-LIF according to the method described by Siri *et al.* [31].

Fatty acids were analyzed by GC as their methyl esters after alkaline hydrolysis of 100 µg LTA in 1N NaOH for 2 h at 37°C.

### MALDI/MS

LTA molecular mass was determined on a Voyager DE-STR MALDI-TOF instrument (PerSeptive Biosystems) using linear mode detection. The matrix used was 2,5-dihydroxybenzoic acid (Sigma) at a concentration of 10 µg.µl^−1^ in a mixture of ethanol/water (1∶1, v/v). 0.5 µl of ShLTA, at a concentration of 10 µg.µl^−1^, were mixed with 0.5 µl of the matrix solution. Mass spectra were recorded in the negative mode using a 350-ns time delay with a grid voltage of 90% of full accelerating voltage (25 kV) and a guide wire voltage of 0.15%.

ShLTA (1 mg) was depolymerized by 48% HF hydrolysis, 48 h at 4°C. After drying under a nitrogen stream, the sample was submitted to a methanol/chloroform/water (1∶2∶3, v/v/v) partition. Aqueous phase, containing glycerophosphate motifs, was analyzed by MALDI/MS and MS/MS on a 4700 Proteomics Analyzer (with TOF/TOF optics, Applied Biosystems, Voyager DE-STR) using the reflectron mode. The matrix used was 2,5-dihydroxybenzoic acid at a concentration of 10 µg.µl^−1^ in a mixture of ethanol/water (1∶1, v/v), 0.1% TFA. 0.3 µl of the aqueous phase, at a concentration of 10 µg.µl^−1^, were mixed with 0.3 µl of the matrix solution. Mass spectra were recorded in the positive mode. Collision-induced dissociation gas type was atmosphere, and the gas pressure was set to medium.

### NMR

NMR spectra were recorded with an Avance DMX500 spectrometer (Bruker) equipped with an Origin 200 SGI using Xwinnmx 2.6. ShLTA was dissolved in D_2_O or H_2_O/D_2_O (9∶1, v/v) (D, 99.97%, Eurisotop) and analyzed in 200×5 mm 535-PP NMR tubes at 298 K. Proton and carbon chemical shifts are expressed in parts per million and referenced relative to internal acetone signals at δ_H_ 2.225 and δ_C_ 34.00 ppm respectively. All details concerning correlation spectroscopy and homonuclear Hartmann-Hahn spectroscopy sequences used and experimental procedures were as previously described [46].

### HEK-TLR2 experiments

The HEK-Blue-2 cell line (InvivoGen), a derivative of HEK293 cells that stably expresses the human TLR2 and CD14 genes along with a NF-κB-inducible reporter system (secreted alkaline phosphatase) was used according to the manufacturer's instruction. Cells were plated at 5×10^4^ cells per well in 96-wells plates and the different stimuli were added at concentrations indicated in the figure legends in the HEK-Blue Detection medium (InvivoGen) that contains a substrate for alkaline phosphatase. Alkaline phosphatase activity was measured after 18 h by reading O.D. at 630 nm. To investigate the CD14 and TLR dependence of stimuli activity, HEK-TLR2 cells were pre-incubated for 30 min at 37°C, before stimuli addition, with various antibodies: 5 µg.ml^−1^ of monoclonal anti-CD14 (clone 134620, R&D Systems), monoclonal anti-TLR1 (Clone H2G2, InvivoGen), monoclonal anti-TLR6 (Clone C5C8, InvivoGen) or an IgG1 isotype control (eBioscience). Anti-LTA (Abcam, Ab12248) antibody was used at a concentration of 5 µg.ml^−1^ and pre-incubated with the different stimuli for 30 min at 37°C before HEK-TLR2 cells addition. Pam_3_CSK_4_ and FSL-1 were purchased from InvivoGen.

### THP1 experiments

THP-1-Blue cell line (InvivoGen), a derivative of THP-1 monocyte/macrophage human cells that stably expresses a NF-κB-inducible reporter system (secreted alkaline phosphatase) was used according to the manufacturer's instructions. Cells were added at 10^5^ cells per well in 96-wells plates in the HEK-Blue Detection medium (InvivoGen) or differentiated with 20 ng.ml^−1^ of PMA for 24 h in RPMI 1640 medium (Lonza) and the different stimuli were added at concentrations indicated in the figure legends. NF-κB activation was measured as described above. IL-6 and TNF-α were assayed in the supernatant by sandwich ELISA using commercially available kits (Diaclone).

### Statistical analysis

Results are expressed as a mean ± SD and were analyzed using the Student's *t* test to determine significant differences between samples.
